# Supplementary material for: Endothelial and Vascular Health: A Tale of Honey, H2O2 and Calcium
Source: Cells. 2021 Apr 30;10(5):1071. doi: 10.3390/cells10051071 (PMC8147193; doi:10.3390/cells10051071)
Supplement: Supplementary file 1 [file cells-10-01071-s001.zip › cells-1200658-supplementary.pdf]

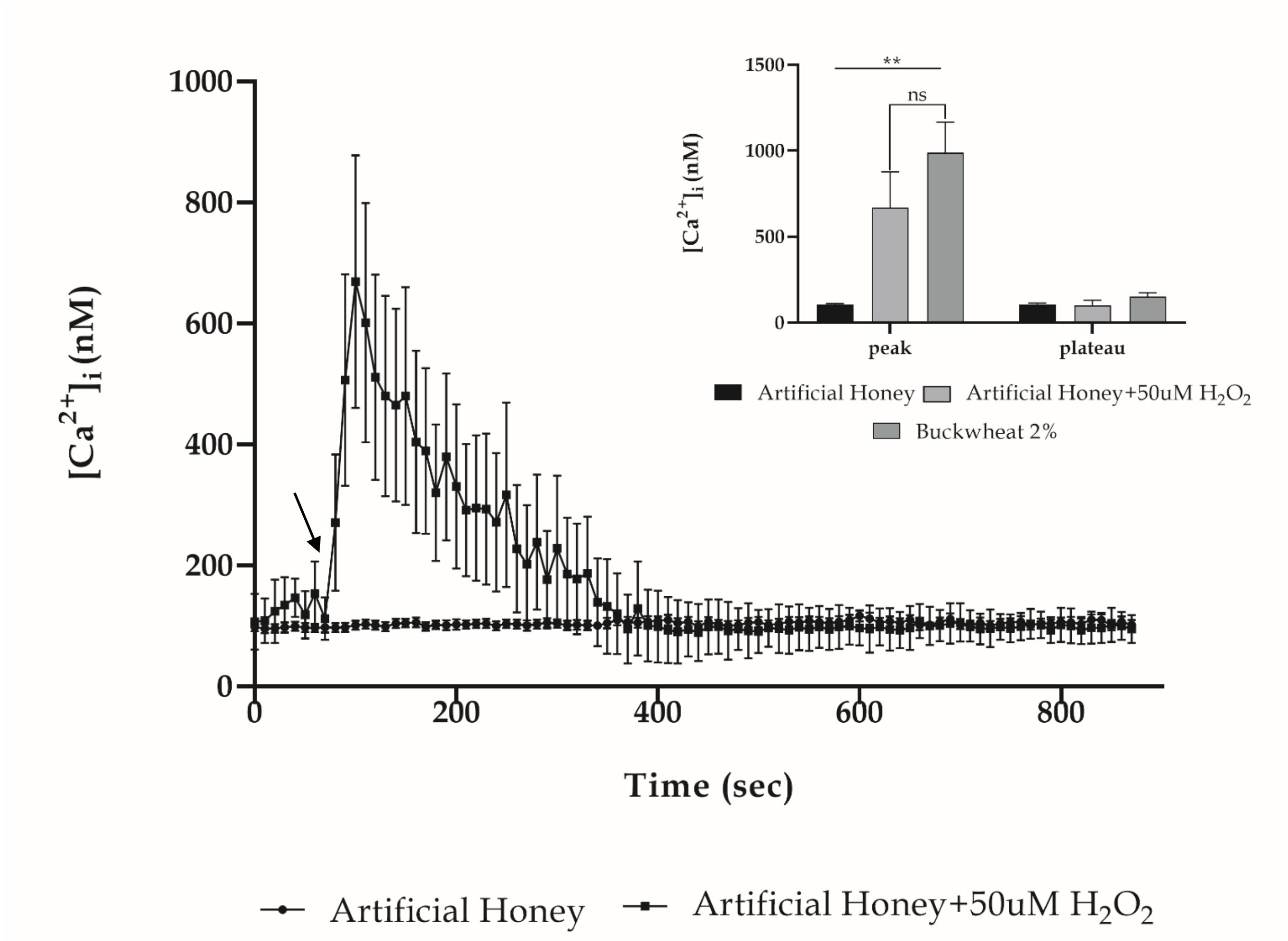

Supplementary Figure S1. Ca^{2+} response in presence of artificial honey. Artificial honey [1], when applied extracellularly, did not boost any variation in $[Ca^{2+}]_i$. after the addition of 50μM H_2O_2 to the artificial honey and it was possible to observe an increase comparable to that observed with 2% BH. Data are means ± s.e.m. of $[Ca^{2+}]_i$ traces measured in different cells. The arrow shows the addition of artificial honey after 60 sec. Number of cells: artificial honey: 40 cells from 3 exp; artificial honey + 50μM H_2O_2: 40 cells from 3 exp. Insert. Mean ± s.e.m. of the Ca^{2+} peak response measured under the indicated treatments or 2% BH. Number of cells as before. Asterisks on bars indicate statistical differences with respect to artificial honey condition determined by a Two-Way ANOVA followed by Tukey's test (** $p < 0.01$).

1. Martinotti, S.; Laforenza, U.; Patrone, M.; Moccia, F.; Ranzato, E. Honey-Mediated Wound Healing: H_2O_2 Entry through AQP3 Determines Extracellular Ca. *Int J Mol Sci* **2019**, *20*, doi:10.3390/ijms20030764.
